# Probing Pharmaceutical Strategies to Promote the Skin Delivery of Asiatic Acid from Hydrogels: Enhancement Effects of Organic Amine Counterions, Chemical Enhancers, and Microneedle Pretreatment

**DOI:** 10.3390/pharmaceutics14112532

**Published:** 2022-11-20

**Authors:** Mingming Li, Qiuyue Wang, Naiying Chen, Sicheng Yao, Xinxing Sun, Peng Quan, Yang Chen

**Affiliations:** 1 Department of Pharmaceutics, School of Pharmacy, China Medical University, Shenyang 110122, China; lmm15638423885@163.com (M.L.); 15765579242@163.com (Q.W.); chennaiying1996@outlook.com (N.C.); loveyaoxiansheng@gmail.com (S.Y.); xinxingsun6820@163.com (X.S.); 2 Department of Pharmaceutics, School of Pharmacy, Shenyang Pharmaceutical University, Shenyang 110016, China

**Keywords:** asiatic acid, hydrogel, counterion, chemical enhancer, microneedle

## Abstract

Asiatic acid (AA) is a pentacyclic triterpene isolated from Centella asiatica, holding great promise for treating a variety of skin disorders. However, the dermal application of AA is limited by its poor solubility and permeability. This study aimed to identify a hydrogel formulation for AA and improve its skin penetration by various penetration enhancement methods. Four kinds of hydrogel bases were selected to prepare the AA hydrogel, in which different organic amines and chemical enhancers were incorporated in combination with microneedle pretreatment. The results showed that AA had good release profiles in the presence of hyaluronic acid as the hydrogel base and organic amines as the counter-ions. Diethylamine and Span 80 could promote drug penetration into the skin, and pretreatment with microneedles could further increase the drug permeability. In conclusion, the optimized hyaluronic acid hydrogel has great potential for use in the topical delivery of AA, and its penetration via the skin can be further improved by different pharmaceutical approaches.

## 1. Introduction

**Figure 1:**
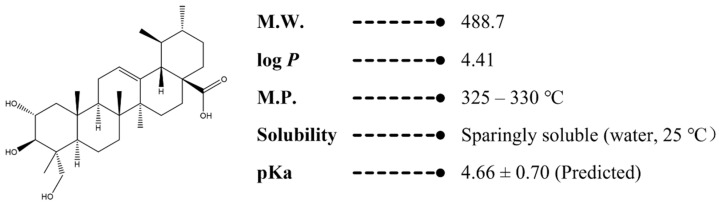
The structure and properties of AA.

Asiatic acid (AA, its structure and properties as shown in Figure 1) is a pentacyclic triterpene isolated from Centella asiatica [1]. It has been reported to have a great variety of pharmacological activities, including anti-oxidative, anti-inflammatory, neuroprotective, gastroprotective, hepatoprotective, cardioprotective, and anticancer properties [2]. AA exhibits great potential in the treatment of many skin disorders [3,4]. For example, A high dose of AA (100 mg/kg, p.o.) produced an anti-psoriatic effect in the imiquimod-induced mouse model through IL-17A and IL-23 pathways [5]. It could also inhibit ultraviolet-A (UVA) induced generation of reactive oxygen species (ROS) and lipid peroxidation, as well as reduce the expression of MMPs and p53, which might be useful for preventing UVA-induced photoaging [6]. Through ROS generation, the change in the Bax/Bcl-2 ratio, and the activation of caspase-3, AA can induce apoptosis of SK-MEL-2 human melanoma cells and may be effective for the treatment of skin cancers [7]. Especially, AA has been identified as the most prominent constituent among the Centella asiatica extractions for wound healing, and its action mechanisms may involve its abilities to increase collagen synthesis, promote extracellular matrix remodeling, and inhibit inflammation [8]. Taken together with these findings, AA appears to be an interesting candidate for dermatological treatment, and in view of the clinical indications of its natural source Centella asiatica, AA has the potential to become an alternative compound approved for wound treatment.

To improve the efficacy of AA for dermal use, topical delivery represents an attractive choice for its effective delivery in the targeted skin, offering many advantages over other drug administration routes, including site-specific delivery, avoidance of first-pass metabolism, noninvasive administration, ease of termination, and good patient compliance [9,10,11]. However, the poor solubility and relatively great molecular weight of AA are the major barricades to its delivery via the skin, and it may be confined to the upper stratum corneum with a prominent barrier function rather than penetrate the deep skin layers [10]. In this regard, penetration enhancement technologies are quite preferable to be used to increase the skin permeability of the impermeable drug. In commercial products, they are adopted either by chemical adjuvants or physical devices, mainly including chemical enhancers, ion pairs, microneedles, and so on [10]. Counterions are particularly effective for the skin delivery of highly lipophilic and ionized drugs to overcome the skin barrier by improving their physicochemical properties dependently on forming ion pairs [12]. Chemical enhancers represent the preferable approach to improve skin drug delivery by interacting with skin constituents to reversibly decrease the barrier resistance [13]. Nowadays, plenty of chemical enhancers have been evaluated to increase drug penetration. Microneedle pretreatment, which depends on the formed transient micropores to bypass the skin barrier, has been widely used in the pharmaceutical and cosmeceutical fields, where AA has shown great potential for use as a drug candidate or a functional ingredient [14]. However, to the best of our knowledge, there have been no reports revealing the skin permeability of AA. Only in a recent investigation was a kind of AA-loaded transfersome developed with the intention of topical use; however, the drug permeability was assessed using the Strat-M^®^ membrane instead of the skin [15]. Therefore, pharmaceutical attempts at skin permeation evaluation and enhancement method development are important and urgent. 

Hydrogels have been proven to be a kind of attractive vehicle for drug delivery in dermatological and cosmetological use due to their desirable biocompatibility and unique properties, such as high-water content and smart drug release [16,17,18,19]. They are usually prepared from natural or synthetic polymers with three-dimensional network structures, which makes them suitable for the incorporation of herb ingredients [20]. For wound healing components such as AA, hydrogels can act as wound dressings to promote healing by mimicking the native skin microenvironment [21]. By changing the polymer types, hydrogels can be designed with different rheological properties and release capacities for satisfying different demands [22,23]. Therefore, hydrogels would be a promising choice for the formulation of AA, which is worthy of in-depth investigation.

**Scheme 1:**
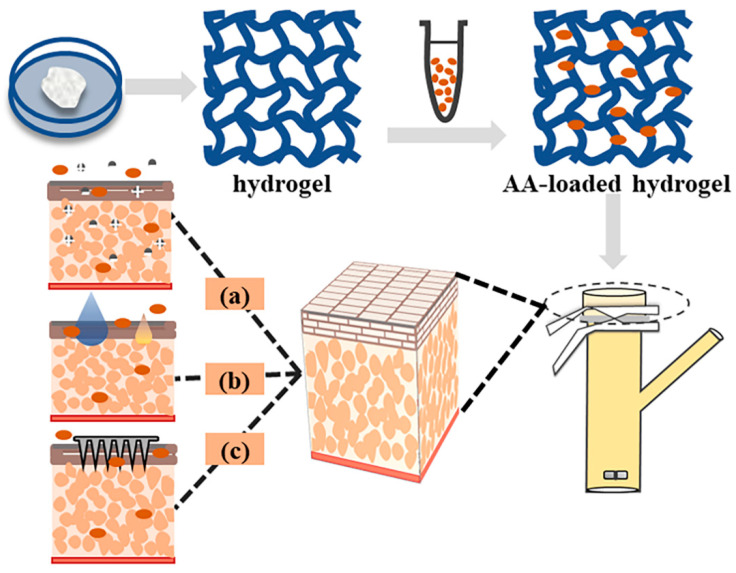
Schematic diagram of the preparation of AA-loaded hydrogel and study of skin penetration promotion in various ways: (**a**) organic amine counterions; (**b**) chemical enhancers; (**c**) microneedles.

In this study, we intended to develop an AA-loaded hydrogel that would be used in our future study to investigate the activity of AA on wound healing. Four types of polymers, including hyaluronic acid (HA), chitosan (CS), poloxamer 407 (P407), and carbomer 940 (C940), were selected to construct the hydrogel bases, and their release profiles toward AA were compared to identify the optimal base for AA. Based on that, different enhancement strategies, including chemical enhancers, counter-ions of organic amines, as well as microneedle pretreatment, were investigated to improve the skin permeability of AA (Scheme 1). The study was expected to help the formulation design of AA for transdermal or dermal application and to understand the action of different enhancement approaches.

## 2. Materials and Methods

### 2.1. Materials

AA, diethylamine (DEtA), triethylamine (TEtA), and ethanolamine (ETA), N-Methylpyrrolidone (NMP) were all purchased from Aladdin Reagent Co., Ltd. (Shanghai, China). HA, CS, C940, P407, oleic acid (OA), polyethylene glycol 400 (PEG 400), glycerol (GL), 1,2-propylene glycol (PG), Azone, Span 80, isopropyl myristate (IPM), L-menthol, and menthyl Lactate (ML), were bought from Shanghai Macklin Biochemical Technology Co., Ltd. (Shanghai, China). Methanol and acetonitrile were chromatographically pure and supplied by Shandong Yuwang Chemical Reagent Co., Ltd. (Yucheng, China). All other reagents used were of analytical or chromatographic grade.

### 2.2. Solubility of AA in Different Solvents

To ensure the sufficient dissolution of AA in hydrogels and maintain the sink condition during the penetration experiments, we determined the solubility of AA in different solvents which might be used for dissolving AA in the preparation of hydrogels or as the receptor liquid in permeation experiments. In different solvents, an excessive amount of AA was added. The mixture was shaken vigorously and then placed in a constant temperature oscillator to equilibrate at 32 ± 1 °C for 72 h. After that, the suspension was filtered through a 0.22 μm filter membrane, and the filtrate was obtained after the discarding of the initial filtrate. The drug content was analyzed by HPLC after appropriate dilution.

### 2.3. Preparation of AA-Loaded Hydrogels

Four types of polymers were selected to prepare the AA-loaded hydrogels, including HA, CS, P407, and C940. First, 0.0580 g AA or its mixture with organic amines (DEtA, TEtA, ETA, NMP) and chemical enhancers were added and dissolved in 1.0000 g PG at 70 °C to get the AA solution (5.8%, *w/w*). Then, the drug solution was added to different hydrogel bases and stirred evenly to obtain the AA-loaded hydrogels with a drug content of 2.0%. For different types of hydrogel bases, they were respectively prepared as follows. HA: 0.0540 g HA was added in 1.35 mL deionized water and then placed at 4 °C overnight for HA swelling to form the hydrogel base. CS: 0.0900 g CS was added and wet in 1.2 mL deionized water, in which 20 μL of acetic acid was subsequently added to form the hydrogel base by stirring. P407: 0.4500 g P407 was added in 1.0 mL deionized water and then placed at 4 °C overnight for swelling to form the hydrogel base. C940: 0.0250 g C940 was added in 0.415 mL deionized water and placed at 4 °C overnight for swelling. After that, a 5% triethanolamine solution of 0.835 mL was added and stirred evenly to form the hydrogel base. Among them, CS and C940 require the addition of pH adjusters to form the hydrogels. All the obtained blank bases were colorless and transparent. After the hydrogel base swelled, the AA solution of PG was added to these hydrogel bases and then stirred evenly to mix well. All prepared AA-loaded hydrogels appeared to be opaque and white in color and could be easily applied to the skin. During the formulation investigations, different base types, HA content, and drug content were changed according to the prescriptions, as listed in Table 1.

For preparing the AA-loaded hydrogels containing organic amines, 9.21 μmol DEtA, ETA, TEtA, or NMP (0.1 mol eq of the drug) were separately added to the AA solution and then mixed with the 3.5% HA hydrogel base to get the preparations. The AA-loaded hydrogel without any organic amine was used as the control to evaluate the effect of counterions on the skin permeation of AA. For preparing the AA-loaded hydrogels containing 5.0% different chemical enhancers, 0.1101 g L-menthol, Azone, OA, Span 80 or ML were separately added in the AA solution along with the DEtA as the preferred organic amine and then mixed with the HA hydrogel base to get the preparations. For investigation of the combined effect of different enhancers, 5.0% L-menthol and 5.0% Span 80 were added into the AA solution and mixed with the HA hydrogel base to get the preparations. Using the DEtA as the preferred organic amine, the AA-loaded hydrogel without any enhancer was used as the control to evaluate the effect of different chemical enhancers on the skin permeation of AA. As for the prescriptions for the microneedle experiment, it is that 9.21 μmol DEtA was added into the AA solution and mixed with a HA hydrogel base to get the formulation. Then, L-menthol as the preferred enhancer and the microneedles of the length of 0.5 mm and 0.75 mm were separately used in combination for experiments. All these prescriptions are listed in Table 2.

### 2.4. Drug Release Experiments

Drug release experiments were carried out with a modified Franz diffusion cell. The cellophane dialysis membrane (pore size, ~100 nm) was placed between the donor cells and the receptor cells, and then AA-loaded hydrogels were added to the donor cells and totally contacted the membrane with a diffusion area of 1.76 cm^2^. PBS containing 3% Tween 80 (*w/v*) of 7.0 mL was added to the receptor cell, which was kept at 32 °C with magnetic stirring at 150 rpm. At 2, 4, 6, 8, 10, and 12 h, 3.5 mL receptor fluid was taken out, and an equivoluminal fresh receptor fluid was added. The sample was centrifuged at 15,000 rpm for 10 min for HPLC analysis. The cumulative release of AA was calculated by Formula (1):

(1) $$Q=\left(C_{i}\times V+\overset{n-1}{\underset{i-1}{\sum }}C_{i-1}\times V_{i}\right)/A$$

*Q*: the cumulative release of the drug; *C_i_*: the concentration of the released liquid of the *i*-th replacement sampling; *C_i_*_−1_: the concentration of the released liquid of the (*i* − 1)-th replacement sampling; *V_i_*: displacement volume of receiving solution; *V*: total volume of release medium (*V* = 7.0 mL); *n*: number of times to replace receiving solution; *A*: effective diffusion area.

### 2.5. Skin Penetration Experiments

Skin penetration experiments were carried out similarly to the release method for the determination of drug penetration through the skin, and the difference was that the semi-permeable membrane was replaced by the excised rat skin. Male Sprague-Dawley (SD) rats (weighing 200–230 g), purchased from SPF (Beijing) Biotechnology Co., Ltd., were first anesthetized with urethane (20%, *w/v*, i.p.). Then, the abdominal hair was successively shaved with an electric clipper and a razor. Full-thickness skin was excised after the rats were sacrificed, and the subcutaneous fat adhering to the skin was surgically trimmed. The obtained skin was cut to the appropriate size, wrapped in aluminum foil, and then immediately stored at −20 °C for a maximum of 2 weeks. Prior to each experiment, the skin was allowed to thaw slowly for subsequent use. The thickness of the rat skin used in our study was 680 ± 50 μm. For microneedle pretreatment, the skin was pierced with an array of microneedles (DRS^®^, 140A) at the predefined depth and force for a certain time. After that, the penetration experiments were carried out accordingly to the above release method. According to the principles of animal ethics, all animal-related experimental procedures were carried out with the approval of the Department of animals of China Medical University.

### 2.6. Extraction of the Drug from Skin

At the end of the skin penetration experiment, drug content in different skin layers was determined. First, the excess hydrogels were removed from the skin surface. The skin was then taken out from the devices, washed with 2.0 mL of fresh water, subsequently dried with filter paper, and weighed. The stratum corneum (SC) of the skin was collected via successive tape striping (20 times) with QJMDM tapes (Hubei Qianjiang Kingphar Medical Material Co., Ltd., Qianjiang, China). The remaining skin was weighed again, and the weight of the stratum corneum was obtained by the difference method. The drug in the stratum corneum and the remaining skin layers were separately extracted via immersion in 3.0 mL and 0.5 mL methanol overnight and ultrasound for 15 min. The obtained suspension was centrifuged at 15,000 rpm and 4 °C for 10 min, and the supernatant was filtered through the 0.22 μm membrane for HPLC analysis of AA content in each skin.

### 2.7. HPLC Analysis of AA

The content of AA was determined with a validated HPLC method. The analysis was performed on an HPLC apparatus equipped with an e2695 pump and a 2489 variable-wavelength UV detector (Waters Corporation, Milford, CT, USA). The separation was carried out on a Diamonsil C18 (2) column (5 μm, 250 mm × 4.6 mm). The mobile phase consisted of a mixture of acetonitrile and purified water (with 0.1% H_3_PO_4_) (62:38, *v/v*). The flow rate was 1.0 mL/min, the column was kept at 30 °C, and the detection wavelength was set to 205 nm.

### 2.8. Determination of Rheological Properties

The dynamic rheological tests of all HA hydrogel samples were carried out by an MCR302 rheometer (Anton Paar, Austria), equipped with Rheoplus software, and CTD 620 gas convection controlled thermostat circulating water bath, appended with cone and plate geometry (CP 25-2, angle 2°). Firstly, to observe the rheological characterization of HA hydrogel when they were used in the skin, these samples were tested at 32 °C, which was the temperature of the skin. Then, the quality of the flow of these hydrogels was tested by a flow curve test. Viscosity (Pa·s) and shear stress (τ) were assessed at the rate of 0.1–100 rad/s. After that, the amplitude sweep test was used to determine viscoelastic behavior. Linear viscoelastic region (LVR) was observed with the measurement of the storage (G′) and loss modulus G″ by keeping the shear stress (Pa) between 0.1 and 100 Pa and the constant frequency of 1 Hz. Lastly, the tests of the storage (G′), loss modulus (G″), and complex viscosity (η) were carried out by frequency sweep measurements in the LVR by setting the angular velocity from 0.1 to 100 rad/s and at 1% constant strain amplitude.

### 2.9. Scanning Electron Microscope (SEM) Analyses

The surface structure of the HA samples was investigated with scanning electron microscopy (S-4800, Hitachi, Ltd., Tokyo, Japan). HA hydrogel samples were freeze-dried at −50 °C, extracted by liquid nitrogen, and then coated with a thin layer of gold for SEM observation. 

### 2.10. Nuclear Magnetic Resonance Spectroscopy (NMR) Analyses

All NMR samples were tested on a 600 MHz Bruker ascend DRX 600 spectrometer, appended with standard Brucker pulse sequences. To obtain samples with that same concentration, 0.0500 g AA with 0.1 mol ratios of DEtA and 0.0050 g AA with equal molar ratios of DEtA were dissolved in DMSO-*d*_6_. Beyond that, 0.0050 g pure AA was dissolved in DMSO-*d*_6_ as a control.

### 2.11. Histological Study of the Microneedle-Treated Skin

The surface of microneedle-treated skin was investigated with the H&E staining method. Firstly, the abdomen of the rats was shaved and then treated with the microneedle (5 N, 6 min, 0.5 mm). At 0 min, 5 min, 10 min, 30 min, and 1 h, the rats were sacrificed, and the skin tissues were separately removed. Lastly, these skin tissues were fixed in 4% paraformaldehyde for 48 h. After paraffin embedding, sectioning, and the H&E staining, the effect of microneedle action on the structure of the skin was observed with an optical microscope.

### 2.12. Statistical Analysis

All experiments were carried out at least in triplicate, and the results were indicated as mean ± SD (n ≥ 3). The difference values of *p* were determined to assess the statistical significance. The value of *p * >  0.05 indicated no significant difference, * *p* < 0.05 indicated a significant difference, and ** *p* < 0.01 indicated a highly significant difference.

## 3. Results and Discussion

### 3.1. Solubility of AA in Different Solvents

For passive skin penetration, drug solubility in vehicles is an important parameter related to drug transport via the skin [24]. Generally, high solubility allows high drug content in preparations, which is beneficial for improving drug permeation flux. Also, the drug should be dissolved well in receptor fluid to ensure that the “sink conditions” and the diffusion process will always be achieved during the penetration experiments [25,26]. However, the solubility of AA is extremely poor, which limits the transdermal/dermal application and assessment. Thus, prior to the cutaneous permeation investigation, the solubility of AA in different solvents was determined, and the results are shown in Table 3. The AA was insoluble in either water or PBS, and it was undetectable in these two media. Among the solvent candidates for preparation, AA has the greatest solubility in PG, which was followed by PEG 400, OA, IPM, and GL. As for the solvent candidates as the receptor fluid in the following experiments, AA has the greatest solubility in PBS containing 3% Tween 80, which was significantly higher than that of PBS containing 3% SDS or 30% PEG 400. Therefore, PG and PBS containing 3% Tween 80 were respectively used as the solvents for dissolving AA during the preparation of its hydrogel and the receptor fluid in the permeation experiments.

### 3.2. Effect of Different Base Types on the Release of AA Hydrogels

**Figure 2:**
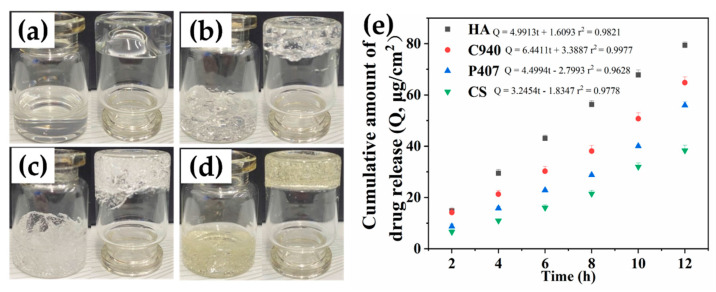
(**a**–**d**) Photographs of the blank hydrogel bases with different polymers, including (**a**) HA, (**b**) C940, (**c)** P407, (**d**) CS; (**e**) effects of base types on the release of AA from the hydrogels. (HA: hyaluronic acid; CS: chitosan; P407: poloxamer 407; C940: carbomer 940).

The type of polymers could significantly affect the drug release and skin penetration from the hydrogels prepared from them [27]. In this study, we selected four kinds of polymers commonly used as hydrogel bases, including HA, CS, P407, and C940, to investigate the effect of polymers on the release profiles of AA. These hydrogels were prepared with the minimum amount of the polymers for gel-forming to control their viscosity, and their pH values were in the neutral range (HA: pH 7.03, CS: pH 5.98, P407: pH 7.51, C940: pH 7.38). The photographs of the blank hydrogels are shown in Figure 2a–d. The result of drug release from different types of hydrogels is shown in Figure 2e. It could be seen that AA had the best release profiles in the case of HA as the hydrogel bases than those observed with C940, CS, and P407. The release of AA was always highest when HA was used (79.38 ± 1.27 μg/cm^2^). It was speculated that the carboxyl groups in HA would provide a mild acid environment to make AA exist in the form of free molecules, which facilitated their diffusion and release [28]. C940 contained neutralized carboxylic acid groups and would not affect the release of AA, which was quite similar to that observed with the HA hydrogels [29]. P407 consisting of oxyethylene and oxypropylene units, had a modest effect on AA release (55.94 ± 1.41 μg/cm^2^), probably due to its H-bonding interaction with the drug [30]. By contrast, AA had the poorest release property when CS was used as the hydrogel base (38.33 ± 2.07 μg/cm^2^), which might be attributed to the electrostatic interactions between the amino group in CS and the carboxyl group in AA. Therefore, it was speculated that the interaction of the drug with the hydrogel base would not benefit its release from the preparations. In addition, zero-order drug release kinetic profiles were noticed for each type of hydrogel with different polymers as the bases. It might be attributed to the relatively high drug loading, which avoided the dose depletion during the release and enabled the drug release rate independent of the drug concentration, and the non-chemical crosslinking structure of hydrogel bases, which also facilitated the drug release. Given the versatile benefits of HA for skin application, including the hydration effect, bioadhesive property, hydrophobic interaction with stratum corneum [31], as well as its beneficial effect on dermatological diseases such as wound healing [32], we selected it as the preferred hydrogel base for further investigations.

### 3.3. Optimization of the Content of HA and AA in the Hydrogels

#### 3.3.1. HA Content

**Figure 3:**
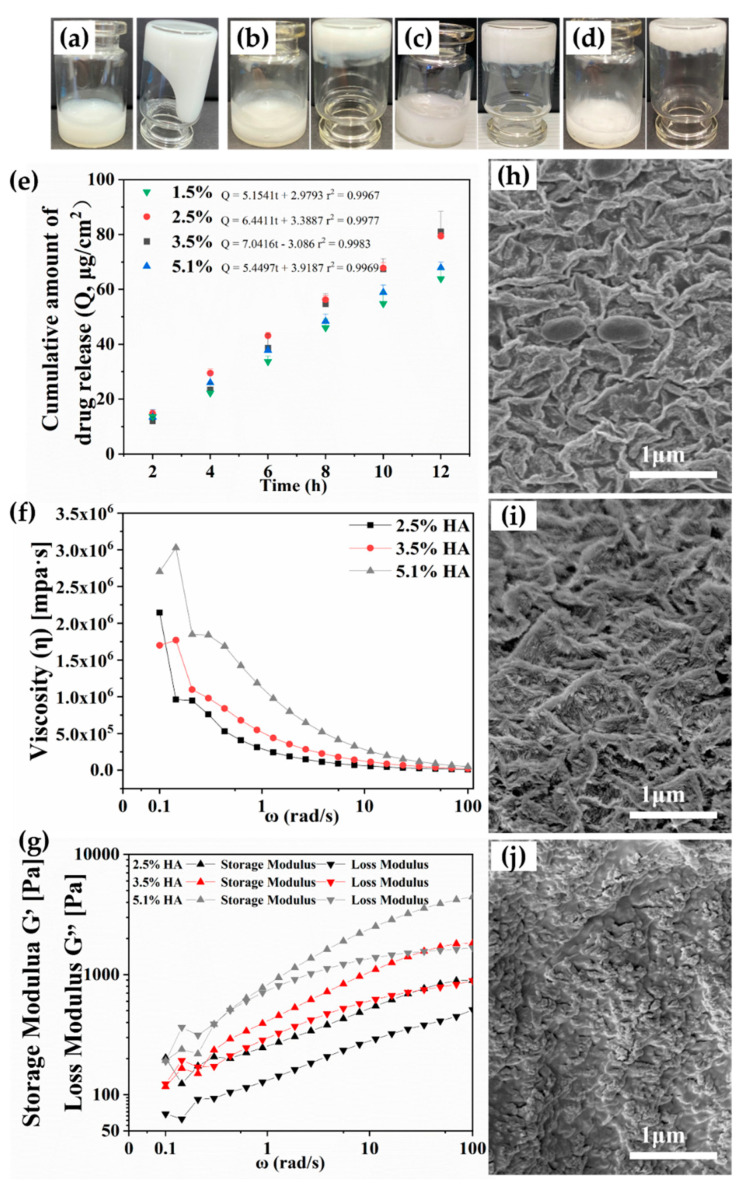
(**a**–**d**) Photographs of the AA-loaded hydrogels of different content of HA: (**a**) 1.5% HA; (**b**) 2.5% HA; (**c**) 3.5% HA; (**d**) 5.1% HA; (**e**) effects of HA content on the release of AA; (**f**) viscosity of hydrogels with different HA content; (**g**) the storage (G′) and loss modulus (G″) of hydrogels with different HA content; (**h**) SEM of hydrogels of 2.5% HA content; (**i**) SEM of hydrogels of 3.5% HA content; (**j**) SEM of hydrogels of 5.1% HA content. (HA: hyaluronic acid).

HA is a natural starting material for constructing hydrogels, and it can form hydrogels via both physical and chemical approaches [33,34]. By comparison with chemical hydrogels, physical hydrogels of HA usually suffer from the weak mechanical property as a result of weak non-covalent interactions of the macromolecular chains, including van der Waals force, hydrophobic forces, H-bonding, ionic interaction, and so on [35]. However, they have no safety issues, which may be caused by unreacted cross-linking agents in chemical hydrogels. Therefore, this study selected the physical method to prepare the HA hydrogels. To improve their rheological property, we optimized the hydrogels by changing the content of HA from 1.5% to 5.1% in the hydrogels. The photographs of the obtained products are shown in Figure 3a–d. When the concentration of HA was low (1.5%), the hydrogels could not be formed due to the excessively high flowability. According to the release result shown in Figure 3e, it could be observed that with the increase of HA content from 1.5% to 2.5%, the release of AA was increased, which was probably because the water affinity of HA promoted the drug release. However, the drug release decreased instead when the HA content further increased from 3.5% to 5.1%, and it was possibly caused by the fact that the network structure was too dense for drug release, which could be seen in the SEM results. For the hydrogels containing 2.5% or 3.5% HA, their drug release amounts were close to each other, and both of them were the greatest, possibly due to their strong hydrogel structure that facilitated the drug release process.

For the hydrogels formed with 2.5%, 3.5%, and 5.1% HA, we investigated their rheological behaviors. As shown in Figure 3f, the viscosity of hydrogels rose with the increase of the HA concentration, and all of them exhibited non-Newtonian properties as their viscosity decreased with the increase of shear force, which was a beneficial factor for the spreadability of hydrogels on the surface of the skin. As shown in Figure 3g, the G′ of hydrogels was also increased with HA content changing from 2.5% to 5.1%, suggesting that the more HA used, the stiffer and more elastic the hydrogels would be. In addition, it could be seen that G′ is constantly higher than G″ for 2.5% and 3.5% HA hydrogel samples, which indicated that they both showed strong gel-like behavior and fair tolerance to external shear force [36]. However, for 5.1% HA hydrogel, G’ showed no obvious difference at low angular frequencies with G″, implying that its stability might not be as good as that of the lower concentration of HA.

SEM images were obtained to determine the microstructure of the freeze-dried hydrogels with different content of HA, and the result is shown in Figure 3h–j; all the hydrogels exhibited porous network structures, which was critical to offer a large specific surface area for solvent uptake and drug diffusion. However, for 5.1% HA, presumably because the content of HA was too much, the network structure of the obtained hydrogel became very dense, which might be unfavorable for drug loading and release. According to the rheological, morphological, and releasing properties, we chose the 3.5% HA as the base of hydrogels for further investigations.

#### 3.3.2. AA Content

**Figure 4:**
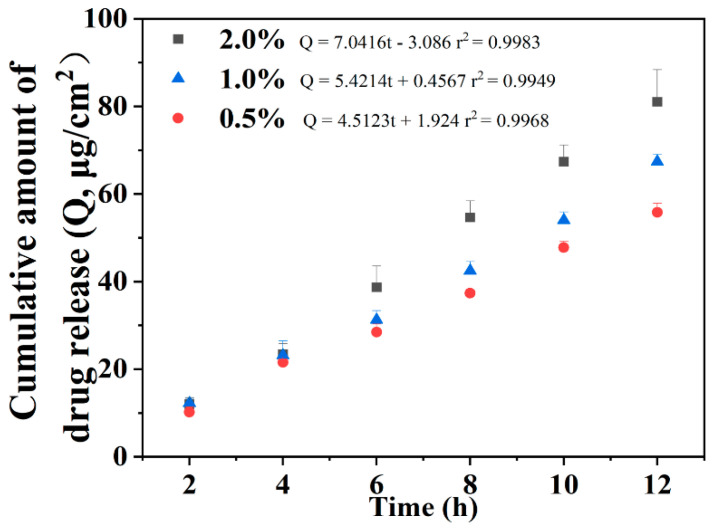
Effects of AA content on the release of AA.

The drug content in hydrogels is another important factor affecting drug release [37], and therefore we investigated the effect of different amounts of AA (0.5%, 1.0%, 2.0%) on its release from hydrogels. The results are shown in Figure 4. It could be seen that the drug release of 0.5%, 1.0%, and 2.0% AA content is 55.81 ± 2.05 μg/cm^2^, 67.36 ± 1.72 μg/cm^2^, 81.05 ± 7.34 μg/cm^2^. This result suggested that the release of AA is a passive diffusion process. Even at 12 h of release, dose depletion was not observed, suggesting that HA was a suitable carrier of AA. We were also informed that as the increase of AA content, the release of AA was increasing gradually. However, a further increase of the AA content would affect the appearance of the drug-carrying hydrogel and cause the AA to be unevenly distributed, and thus the optimal drug loading was selected as 2.0%.

### 3.4. Penetration of AA from Hydrogels into the Skin

The human skin is the best choice for use in percutaneous studies; however, it is not always available and may also bring ethical problems. Additionally, the large variation in the permeability of human skin is also a point of concern. As a potential substitute, rat skin is the most commonly used to estimate the skin permeability of various drugs for the development of skin-related pharmaceutical formulations due to its accessibility and easy handling [38]. Therefore, in the present study, we selected rat skin as the permeation barrier for assessing the skin permeability of AA and the effects of different enhancement methods.

#### 3.4.1. Effects of Organic Amine Counterions

**Figure 5:**
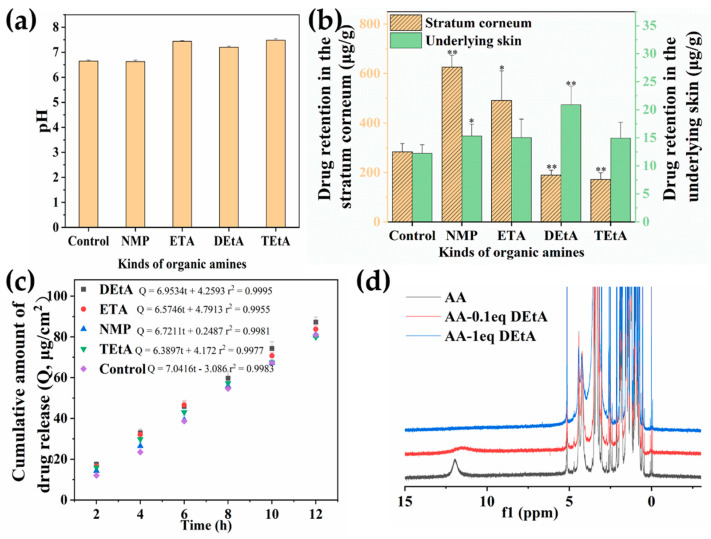
(**a**) The pH of the prescriptions with different kinds of organic amines; (**b**) effects of different organic amines on the content in the stratum corneum and remaining skin compared with the control group (AA-loaded hydrogel without any organic amine); (**c**) effects of different organic amines on the release of AA; (**d**) ^1^H-NMR of AA, AA-DEtA (1:0.1), AA-DEtA (1:1). (NMP: N-Methylpyrrolidone; ETA: ethanolamine; DEtA: diethylamine; TEtA: triethylamine; AA: Asiatic acid; *: *p* < 0.05; **: *p* < 0.01).

The skin permeability of ionizable drugs like AA is not only affected by their physicochemical properties but also by counterions [39]. Unlike covalent modifications, the incorporation of counterions could reversibly modify the physicochemical property of drugs to improve their skin penetration without changing the pharmacological action [40]. In this study, we selected four different types of organic amines, including DEtA, TEA, TEtA, and NMP, to investigate the effect of organic amine counterions on the skin penetration of AA from hydrogels. The pH of the prescriptions is shown in Figure 5a.

The experimental permeation results are shown in Figure 5b. It could be observed that NMP (*p* < 0.01) and ETA (*p* < 0.05) could significantly increase the drug distribution in the stratum corneum, while all the organic amines improved drug retention in the underlying skin, among which the DEtA (*p* < 0.01) exhibited the greatest enhancement effect. We also investigated the effect of organic amines on the release of AA. According to the result shown in Figure 5c, the incorporation of organic amines only slightly increased the drug release from the hydrogel. Therefore, the main cause of their enhancement effect would be attributed to their formation of ion pairs with the drug, which had been widely identified as a potential approach to improve the skin penetration of acidic or basic drugs with high lipophilicity [41,42]. The counter ions could electrostatically interact with the ionized AA, enabling the ‘neutral’ ion pair easier to partition from aqueous bases into an organic stratum corneum layer [41]. The obvious enhancement effect of NMP and ETA on skin penetration in the stratum corneum was presumably due to the hydrogen-bond forming ability of NMP and ETA, which could facilitate their interaction and drug miscibility with the stratum corneum [43]. Once the ion pair entered into the viable epidermis, it would be dissociated and release the drug [44]. The greatest effect of DEtA might be due to its small steric hindrance and the highest alkalinity. Taking the DEtA as an example, we confirmed the formation of ion pair between it and AA via the ^1^H-NMR investigation. The ^1^H-NMR spectra of AA, AA-0.1 mol eq DEtA and AA-1 mol eq DEtA are shown in Figure 5d. For AA, the signal of COOH was detected at 11.96 ppm. Compared with AA, this signal was weakened for the sample of AA-0.1 mol eq DEtA, which proved that the H from COOH of the drug partially interacted with the N from DEtA. As for AA-1 mol eq DEtA, the H signal of COOH disappeared because the N from DEtA interacted with H from COOH thoroughly. Although the 1 mol eq DEtA provided a stronger interaction with the drug, its addition would make the hydrogel beyond the neutrality (pH > 8), which would lead to irritation and damage to the skin. Therefore, we selected the 0.1 mol eq DEtA to improve the deep penetration of AA in the skin from its hydrogels. 

#### 3.4.2. Effects of Chemical Enhancers

**Figure 6:**
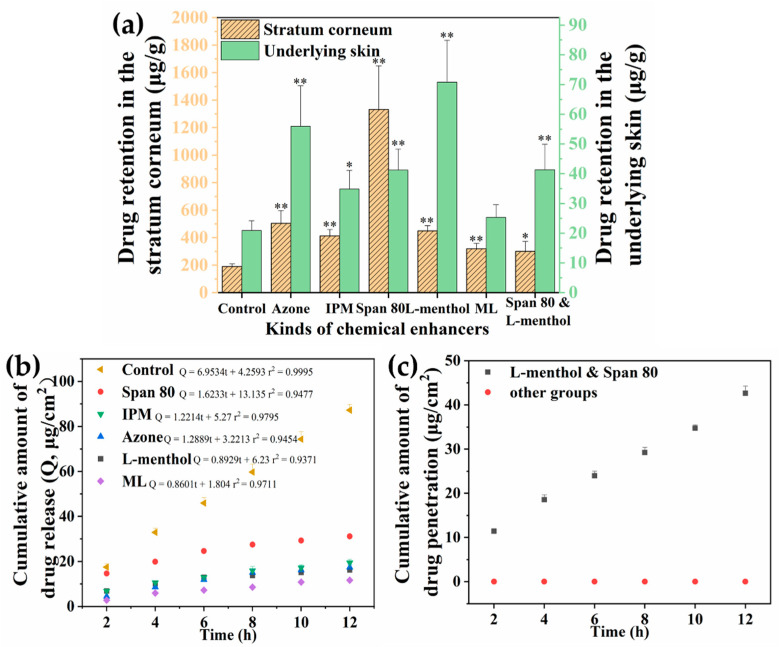
(**a**) Effects of chemical enhancers on the AA content in the stratum corneum and the remaining skin; (**b**) effects of chemical enhancers on the release of AA; (**c**) effects of chemical enhancer combination (L-menthol and Span 80) on the AA content in receptor fluid compared with other groups (including the control group of 0.1 mol eq DEtA added AA-loaded hydrogel and other groups including the hydrogels containing only one kind of enhancers, i.e., Azone, IPM, Span 80, L-menthol and ML (IPM: isopropyl myristate; ML: menthyl Lactate; *: *p* < 0.05; **: *p* < 0.01).

Chemical enhancers play an important role in promoting the absorption of transdermal drugs and are commonly used to promote the skin delivery of drugs [45,46]. The study selected five types of chemical enhancers, including Azone, IPM, Span 80, L-menthol, and ML, to compare their different effects on the skin permeation of AA. The result is shown in Figure 6a. It was found that almost all the enhancers promoted drug retention both in the stratum corneum and the remaining skin. In the case of the stratum corneum, the enhancement effect could be ranked in descending order as Span 80, Azone, IPM, ML, and L-menthol, and all of them were significantly better than that of the control group (*p* < 0.01). Last but not least, AA was not detected in the receptor cells of all chemical enhancer groups in our study. As a non-ionic and lipophilic surfactant, Span 80 was reported to be only deposited in the lipophilic stratum corneum after topical application [47], thus improving the drug distribution in the stratum corneum due to its solubilization effect as a surfactant. While for the remaining skin layer, this order was observed as L-menthol, Azone, Span 80, IPM, and ML, and the drug content of L-menthol was significantly higher than that of the control group (*p* < 0.01). The possible mechanism for L-menthol might be that it had strong hydrogen bonding capability and could compete for the hydrogen bonding sites present in the stratum corneum, making the drug get rid of the interaction with the stratum corneum components and transport into the deep skin layers [48,49]. Apart from the action on the skin, chemical enhancers might also change drug release to influence the skin permeation of the drug. To elucidate this question, we carried out the release experiments of the AA hydrogels containing various kinds of chemical enhancers. The result is shown in Figure 6b. It was surprising to find that the addition of enhancers remarkably reduced the drug release, which could be attributed to their increase in the lipophilicity of the bases, thus impeding the release of the lipophilic AA. In addition, for L-menthol, which could easily form hydrogen bonds with HA, it may be more difficult for AA to release from the dense bases.

As the most effective components, respectively, on the stratum corneum and the remaining skin layers, Span 80 and L-menthol were also investigated for their combined enhancement effect. However, they did not provide a synergistic or combined efficacy, as the enhancement effect was not as good as that when they were used alone. Interestingly, a considerable amount of drug permeation through the skin was noted in the receptor fluid when Span 80 and L-menthol were used together, which was shown in Figure 6c. The possible reason might be that the two enhancers acted at different sites in the skin, including both the stratum corneum and the underlying skin layers, overcoming the barrier function of the whole skin to transport the drug into the receptor fluid, which might reduce drug retention in the skin. Considering that AA might require penetration in the deep skin layers to exert its local treatment effect, the study selected L-menthol as the preferred chemical enhancer for the formulation of AA hydrogels.

#### 3.4.3. Effects of Microneedle Pretreatment

**Figure 7:**
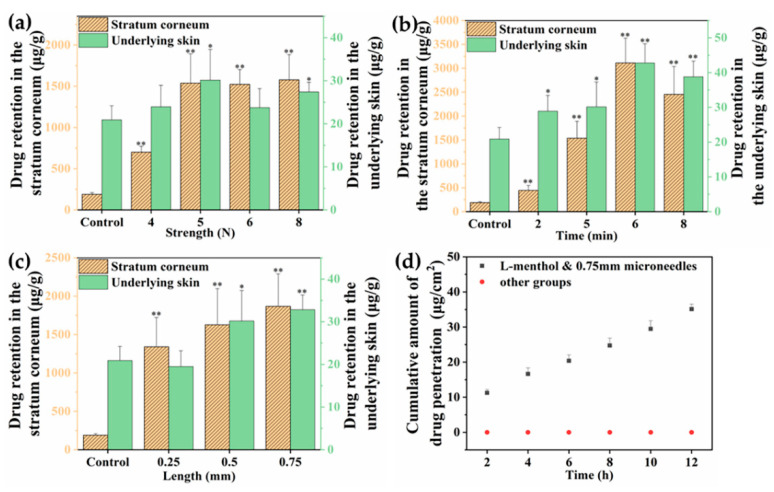
The content of AA in the stratum corneum and the remaining skin with the action of microneedles of (**a**) microneedles of strength; (**b**) microneedles of time; (**c**) microneedles of length; (**d**) the AA content in receptor cells in the group of the combination of L-menthol and microneedle (5 N, 6 min, 0.75 mm) and other groups including various microneedle of strength, time and length and the combination of L-menthol and microneedle (5 N, 6 min, 0.5 mm) (*: *p* < 0.05; **: *p* < 0.01).

As a novel enhancement technology, microneedles depend on single or multiple needle-like structures to pierce the stratum corneum and create multiple microchannels in a minimally invasive manner to improve the permeability of drugs [50]. The microneedles could be applied in a variety of forms, among which the microneedle pretreatment represents a clinically acceptable alternative [51]. In this study, we introduced this strategy to improve the local delivery of AA from hydrogels via changing application conditions, including force size, treatment time, and needle length. The experimental results are shown in Figure 7. Firstly, it can be seen from Figure 7a that with the increase of the force, the content of AA in the stratum corneum gradually increased. In addition, the drug content of 4 N (*p* < 0.01), 5 N (*p* < 0.01), 6 N (*p* < 0.01), and 8 N (*p* < 0.01) groups were significantly better than the control group, while the drug content in the remaining skin layers was a little parabolic with the magnitude of the force. When the force was less than 5 N, the drug content in the skin rose with the increase of the force, while when the force was greater than 5 N, it decreased with the increase of the force. It might be because the skin was excessively pressed, making the state of the skin change, and it becomes difficult for the drug to penetrate. Furthermore, it can be seen from Figure 7b that there was a trend that the content of AA in both the stratum corneum and the remaining skin layers was generally increased with the prolongation of the action time. Above all, the drug content in the stratum corneum of 2 min was higher than the control group (*p* < 0.01), and for the 5 min, 6 min, and 8 min groups, the differences were more pronounced (*p* < 0.01). It was possible because the longer the microneedle action time, the more obvious the holes left by the microneedles on the surface of the skin, which make it easier for the drug to enter the skin through the holes, thereby increasing the intradermal drug content. Lastly, it could be seen from Figure 7c that with the increase of the length of microneedles within a certain range, the drug content of the stratum corneum and the remaining skin layers was gradually increased, and the two are in a positive proportional relationship. What’s more, the drug content in the stratum corneum of all groups was significantly higher than that of the control group (*p* < 0.01). It indicated that the increase in the length of microneedles could promote the entry of AA into the skin to a certain extent. It was possible because the longer the microneedle penetrated the skin, the more favorable it would be for the drug to pass through the stratum corneum and into the deeper layers. In addition, we observed that AA was not detected in the receptor cells of all of the above experimental groups, probably due to the troublesome physicochemical properties of AA which was unbeneficial for its delivery through the micropores formed by the microneedles.

After that, the microneedle pretreatment was used together with the best enhancer, L-menthol, to further enhance the skin penetration of AA. Under the action of 0.5 mm microneedle and L-menthol, there was no improvement for the drug deposition in stratum corneum (228.09 ± 40.54 μg/g) or underlying skin (37.50 ± 7.15 μg/g). A further increase in the microneedle depth (0.75 mm) combined with L-menthol only significantly enhanced the drug retention in stratum corneum (344.81 ± 49.33 μg/g) instead of underlying skin (40.26 ± 3.39 μg/g). Disappointingly, we also found that the combination of 0.75 mm microneedle and L-menthol produced drug penetration into the fluid receptor, which was shown in Figure 7d. It might be because the holes generated by microneedles enabled the L-menthol to drag the drug penetrating across the whole skin layers and reach the receptor fluid [52]. By comparison, this phenomenon was not observed by the single use of any microneedle, any enhancer, and even the combination of 0.5 mm microneedle and L-menthol. Therefore, we inferred that the microneedle could be used to improve the topical penetration of AA without the need for combined use of chemical enhancers, which could increase the risk of systemic drug permeation when the long microneedles (e.g., 0.75 mm) were applied.

### 3.5. Histological Study of the Microneedle-Treated Skin

**Figure 8:**
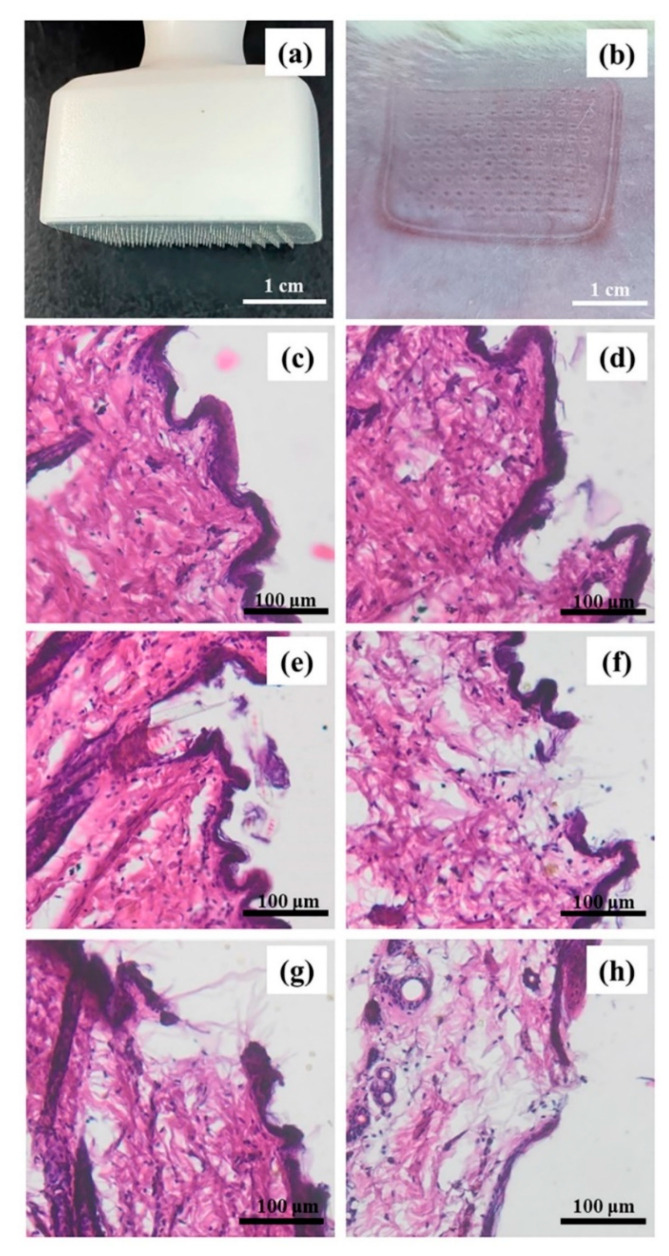
(**a**) The microneedles used in the study; (**b**) the skin treated by microneedles; (**c**–**h**) light microscope observation of H&E strained longitudinal sections to identify the microchannels in the skin tissues after being treated by microneedle at different times: (**c**) control group; (**d**) 0 min; (**e**) 5 min; (**f**) 10 min; (**g**) 30 min; (**h**) 1 h.

In our study, we investigate the state of microneedle-treated skin using the H&E staining method in order to observe changes affected by the action of microneedles. The microneedle used in our experiment is shown in Figure 8a, the surface of the rat skin after microneedle treatment is shown in Figure 8b, and the H&E results are shown in Figure 8c–h. From the stained skin tissue, it could be seen that compared with the control group (Figure 8c), the microneedle penetrated the stratum corneum and viable epidermis to reach the papillary dermis (PD) layer (the layer located directly beneath the viable epidermis) [53]. As exhibited in Figure 8d,e, it hadn’t changed much within 5 min. However, at 10 min (Figure 8f), the skin beneath the stratum corneum gradually recovered, presumably because of the elasticity of the skin and the self-healing ability of the organism. The longer the time, the better the skin recovered, as shown in Figure 8g. Last but not least, the stratum corneum was not completely recovered after 1 h (Figure 8h), allowing a prolonged enhancement effect for skin penetration. From the above results, it could be seen that microneedle didn’t destroy the deep tissues of the skin where the nerve was abundant; thus, trauma was minimized, and the patient’ suffering could be greatly reduced.

## 4. Conclusions

In this study, we developed a novel kind of hydrogel for AA and investigated different pharmaceutical approaches to improve the dermal delivery of AA. HA of 3.5% content was used as the preferred hydrogel base, according to the drug release profile, as well as morphological rheological properties. The counterion that provided the greatest penetration enhancement effect of AA was AA-DEtA, and the optimal chemical enhancer offering the highest drug penetration in deep skin layers was L-menthol. The optimized application scheme of microneedle pretreatment was determined as 6 min of treatment time, 5 N of force, and a needle length of 0.5 mm.

In summary, all these approaches could increase drug retention in the skin when they are used alone. Different types of counterions and chemical enhancers may provide specific enhancement effects towards drug retention in stratum corneum or underlying skin layers. Interestingly, the study found that the combination of enhancer-microneedles or enhancer-enhancer could produce transdermal absorption of the drug. For topical delivery of AA, the most appropriate strategy might be the cooperation of DEtA and the best chemical enhancer of L-menthol, which provided the greatest drug content of 70.80 ± 14.07 μg/g in the deep skin layers. The study could facilitate the design and development of the topical drug delivery system for AA and other drugs with similar physicochemical properties. 

## Figures and Tables

**Table 1:** Prescriptions of AA-loaded hydrogels with different polymers as bases.

Experiments	Polymer		H_2_O (%)	AA-PG Solution * (%)		pH Regulators ^#^ (%)	pH
	Types	Amount (%)		PG (%)	AA (%)		
Base Types	HA	2.5	62.3	33.2	2.0	/	6.75
	CS	4.4	58.9	33.7	2.0	1.0 HAc	5.93
	C940	1.3	21.1	33.1	2.0	42.5 TEA (5% aq.)	7.21
	P407	20.1	44.8	33.1	2.0	/	7.37
HA content	2.5% HA	2.5	62.3	33.2	2.0	/	6.75
	3.5% HA	3.5	61.3	33.2	2.0	/	6.65
	5.1% HA	5.1	59.6	33.3	2.0	/	6.53
Drug content	3.5% HA	3.5	61.3	34.7	0.5	/	7.27
	3.5% HA	3.5	61.3	34.2	1.0	/	6.98
	3.5% HA	3.5	61.3	33.2	2.0	/	6.65

* The AA-PG solution (2.9%, *w/w*) was used to prepare 1.0% AA-loaded hydrogels, the AA-PG solution (1.45%, *w/w*) was used to prepare 0.5% AA-loaded hydrogels, and the AA-PG solution (5.8%, *w/w*) was used to prepare other hydrogels. ^#^ “/” means that no such agents were added. (HA: hyaluronic acid; CS: chitosan; AA: Asiatic acid; PG: 1,2-propylene glycol; AA-PG: the 1,2-propylene glycol solution that AA dissolved).

**Table 2:** Prescriptions of AA-loaded hydrogels containing organic amines and/or chemical enhancers.

Experiments	Organic Amine		Chemical Enhancer		HA (%)	H_2_O (%)	AA-PG (%)	
	Types	Amount (mol eq of AA)	Types	Amount (%)			PG	AA
Organic amine	* DEtA	0.1	/	0	3.5	61.3	33.2	2.0
	TEtA	0.1	/	0	3.5	61.3	33.2	2.0
	NMP	0.1	/	0	3.5	61.3	33.2	2.0
	ETA	0.1	/	0	3.5	61.3	33.2	2.0
Chemical enhancer	DEtA	0.1	Azone	5.0	3.5	61.3	33.2	2.0
	DEtA	0.1	IPM	5.0	3.5	61.3	33.2	2.0
	DEtA	0.1	Span 80	5.0	3.5	61.3	33.2	2.0
	DEtA	0.1	L-menthol	5.0	3.5	61.3	33.2	2.0
	DEtA	0.1	ML	5.0	3.5	61.3	33.2	2.0
Enhancer combination	DEtA	0.1	L-menthol	5.0	3.5	61.3	33.2	2.0
			Span 80	5.0				
Microneedle pretreatment	DEtA	0.1	/	0	3.5	61.3	33.2	2.0
Microneedle & enhancers	DEtA	0.1	L-menthol	5.0	3.5	61.3	33.2	2.0

* DEtA: diethylamine; TEtA: triethylamine; NMP: N-Methylpyrrolidone; ETA: ethanolamine; IPM: isopropyl myristate; ML: menthyl Lactate; AA: Asiatic acid; PG: 1,2-propylene glycol; AA-PG: the 1,2-propylene glycol solution that AA dissolved.

**Table 3:** Solubility of AA in different solvents (n = 3).

Solvents for Preparation	Solubility (mg/mL)	Solvents for Receptor Fluid	Solubility (mg/mL)
Water	/	PBS	/
IPM	112.41 ± 22.04	3% Tween 80/PBS	644.24 ± 49.54
PG	5365.38 ± 333.99	3% SDS/PBS	312.75 ± 37.65
* PEG 400	3877.34 ± 44.72	30% PEG 400/PBS	128.90 ± 17.08
OA	522.03 ± 16.19		
GL	89.84 ± 12.35		

* PEG 400: polyethylene glycol 400; IPM: isopropyl myristate; PG: 1,2-propylene glycol; OA: oleic acid; GL: glycerol; PBS: phosphate buffer saline; SDS: sodium dodecyl sulfate.

## Data Availability

Not applicable.

## References

[1] Razali N.N.M., Ng C.T., Fong L.Y. (2019). Cardovascular Protective Effects of Centella asiatica and Its Triterpenes: A Review. Planta Med..

[2] Meeran M.F.N., Goyal S.N., Suchal K., Sharma C., Patil C.R., Ojha S.K. (2018). Pharmacological Properties, Molecular Mechanisms, and Pharmaceutical Development of Asiatic Acid: A Pentacyclic Triterpenoid of Therapeutic Promise. Front. Pharmacol..

[3] Park K.S. (2021). Pharmacological Effects of Centella asiatica on Skin Diseases: Evidence and Possible Mechanisms. Evid. Based Complement. Altern. Med..

[4] Lv J., Sharma A., Zhang T., Wu Y., Ding X. (2018). Pharmacological Review on Asiatic Acid and Its Derivatives: A Potential Compound. SLAS Technol..

[5] Somboonwong J., Kankaisre M., Tantisira B., Tantisira M.H. (2012). Wound healing activities of different extracts of Centella asiatica in incision and burn wound models: An experimental animal study. BMC Complement. Altern. Med..

[6] Kukula O., Kirmizikan S., Tiryaki E.S., Cicekli M.N., Gunaydin G. (2022). Asiatic acid exerts an anti-psoriatic effect in the imiquimod-induced psoriasis model in mice. Immunopharmacol. Immunotoxicol..

[7] Lee Y.S., Jin D.-Q., Beak S.-M., Lee E.-S., Kim J.-A. (2003). Inhibition of ultraviolet-A-modulated signaling pathways by asiatic acid and ursolic acid in HaCaT human keratinocytes. Eur. J. Pharmacol..

[8] Park B.C., Bosire K.O., Lee E.-S., Lee Y.S., Kim J.-A. (2005). Asiatic acid induces apoptosis in SK-MEL-2 human melanoma cells. Cancer Lett..

[9] Anantrao J.H., Nath P.A., Nivrutti P.R. (2021). Drug Penetration Enhancement Techniques in Transdermal Drug Delivery System: A Review. J. Pharm. Res. Int..

[10] Chen Y., Feng X., Meng S. (2019). Site-specific drug delivery in the skin for the localized treatment of skin diseases. Expert Opin. Drug Deli..

[11] Alkilani A.Z., Nasereddin J., Hamed R., Nimrawi S., Hussein G., Abo-Zour H., Donnelly R.F. (2022). Beneath the skin: A Review of Current Trends and Future Prospects of Transdermal Drug Delivery Systems. Pharmaceutics.

[12] Hui M., Quan P., Yang Y., Fang L. (2016). The effect of ion-pair formation combined with penetration enhancers on the skin permeation of loxoprofen. Drug Deliv..

[13] Williams A.C., Barry B.W. (2012). Penetration enhancers. Adv. Drug Deliv. Rev..

[14] Shin C.I., Kim M., Kim Y. (2019). Delivery of Niacinamide to the Skin Using Microneedle-Like Particles. Pharmaceutics.

[15] Opatha S.A.T., Titapiwatanakun V., Boonpisutiinant K., Chutoprapat R. (2022). Preparation, Characterization and Permeation Study of Topical Gel Loaded with Transfersomes Containing Asiatic Acid. Molecules.

[16] Kesharwani P., Bisht A., Alexander A., Dave V., Sharma S. (2021). Biomedical applications of hydrogels in drug delivery system: An update. J. Drug Deliv. Sci. Technol..

[17] Ahsan A., Tian W.-X., Farooq M.A., Khan D.H. (2020). An overview of hydrogels and their role in transdermal drug delivery. Int. J. Polym. Mater. Polym. Biomater..

[18] Croisfelt F.M., Tundisi L.L., Ataide J.A. (2019). Modified-release topical hydrogels: A ten-year review. J. Mater. Sci..

[19] Ullah A., Lim S.I. (2022). Bioinspired tunable hydrogels: An update on methods of preparation, classification, and biomedical and therapeutic applications. Int. J. Pharm..

[20] Lai W.-F., Rogach A. (2017). Hydrogel-Based Materials for Delivery of Herbal Medicines. ACS Appl. Mater. Interfaces.

[21] Tavakoli S., Klar A.S. (2020). Advanced Hydrogels as Wound Dressings. Biomolecules.

[22] Wróblewska M., Słyż J., Winnicka K. (2019). Rheological and textural properties of hydrogels, containing sulfur as a model drug, made using different polymers types. Polimery.

[23] Lacroce E., Rossi F. (2022). Polymer-based thermoresponsive hydrogels for controlled drug delivery. Expert Opin. Drug Deliv..

[24] Sato M., Mitsui N., Hasegawa T., Sugibayashi K., Morimoto Y. (2021). Potential usefulness of solubility index for prediction of the skin permeation rate of 5-ISMN from pressure-sensitive adhesive tape. J. Control Release.

[25] Biondo N.E., Argenta D.F., Rauber G.S., Caon T. (2021). How to define the experimental conditions of skin permeation assays for drugs presenting biopharmaceutical limitations? The experience with testosterone. Int. J. Pharm..

[26] Cilurzo F., Musazzi U.M., Franzé S., Fedele G., Minghetti P. (2018). Design of in vitro skin permeation studies according to the EMA guideline on quality of transdermal patches. Eur. J. Pharm. Sci..

[27] Djekic L., Martinović M., Dobričić V., Čalija B., Medarević Đ., Primorac M. (2019). Comparison of the Effect of Bioadhesive Polymers on Stability and Drug Release Kinetics of Biocompatible Hydrogels for Topical Application of Ibuprofen. J. Pharm. Sci..

[28] Tiwari S., Bahadur P. (2019). Modified hyaluronic acid based materials for biomedical applications. Int. J. Biol. Macromol..

[29] Zhang F., Lubach J., Na W., Momin S. (2016). Interpolymer Complexation Between Polyox and Carbopl, and Its Effect on Drug Release from Matrix Tablets. J. Pharm. Sci..

[30] Abdeltawab H., Svirskis D., Sharma M. (2020). Formulation strategies to modulate drug release from poloxamer based in suit gelling systems. Expert Opin. Drug Deliv..

[31] Zhu J., Tang X., Jia Y., Ho C.-T., Huang Q. (2020). Applications and delivery mechanisms of hyaluronic acid used for topical/transdermal delivery—A review. Int. J. Pharm..

[32] Wang L., Li J., Xiong Y., Wu Y., Yang F., Guo Y., Chen Z., Gao L., Deng W. (2021). Ultrashort Peptides and Hyaluronic Acid-Based Injectable Composite Hydrogels for Sustained Drug Release and Chronic Diabetic Wound Healing. ACS Appl. Mater. Interfaces.

[33] Bao Z., Xian C., Yuan Q., Liu G., Wu J. (2019). Natural Polymer-Based Hydrogels with Enhanced Mechanical Performances: Preparation, Structure, and Property. Adv. Healthc. Mater..

[34] Xu X., Jha k.A., Harrington A.D., Carson M.C.F., Jia X. (2012). Hyaluronic Acid-Based Hydrogels: From a Natural Polysaccharide to Complex Networks. Soft Matter..

[35] Trombino S., Servidio C., Curcio F., Cassano R. (2019). Strategies for Hyaluronic Acid-Based Hydrogel Design in Drug Delivery. Pharmaceutics.

[36] Zhang R., Li X., He K., Sheng X., Deng S., Shen Y., Chang G., Ye X. (2017). Preparation and properties of redox responsive modified hyaluronic acid hydrogels for drug release. Polym. Adv. Technol..

[37] Sivasankaran S., Jonnalagadda S. (2022). Levonorgestrel loaded biodegradable microparticles for injectable contraception: Preparation, characterization and modelling of drug release. Int. J. Pharm..

[38] Takeuchi H., Mano Y., Terasaka S., Sakurai T., Furuya A., Urano H., Sugibayashi K. (2011). Usefulness of Rat Skin as a Substitute for Human Skin in the in Vitro Skin Permeation Study. Exp. Anim..

[39] Cilurzo F., Minghetti P., Alberti E., Gennari C.G.M., Pallavicini M., Valoti E., Montanari L. (2010). An Investigation into the Influence of Counterion on the RS-Propranolol and S-Propranolol Skin Permeability. J. Pharm. Sci..

[40] Geng S., Liu X., Xu H. (2016). Clarithromycin ion pair in a liposomal membrane to improve its stability and reduce its irritation caused by intravenous administration. Expert Opin. Drug Deliv..

[41] Cristofoli M., Hadgraft J., Lane M.E., Sil B.C. (2022). A preliminary investigation into the use of amino acids as potential ion pairs for diclofenac transdermal delivery. Int. J. Pharm..

[42] Song H., Liu C., Ruan J., Yang D., Zhong T., Liu Y., Fang L. (2022). Effect of the combination of permeation enhancer and ion-pairs strategies on transdermal delivery of tofacitinib. Int. J. Pharm..

[43] Yang D., Liu C., Ding D., Quan P., Fang L. (2021). The molecular design of drug-ionic liquids for transdermal drug delivery: Mechanistic study of counterions structure on complex formation and skin permeation. Int. J. Pharm..

[44] Zhao H., Liu C., Quan P., Wan X., Shen M., Fang L. (2017). Mechanism study on ion-pair complexes controlling skin permeability: Effect of ion-pair dissociation in the viable epidermis on transdermal permeation of bisoprolol. Int. J. Pharm..

[45] Prausnitz M.R., Langer R. (2008). Transdermal drug delivery. Nat. Biotechnol..

[46] Kováčik A., Kopečná M., Vávrová K. (2020). Permeation enhancers in transdermal drug delivery: Benefits and limitations. Expert Opin. Drug Deliv..

[47] Ruan J., Chao L., Wang J., Zhong T., Quan P., Fang L. (2022). Efficacy and safety of permeation enhancers: A kinetic evaluation approach and molecular mechanism study in the skin. Int. J. Pharm..

[48] Chen L., Ma L., Yang S., Wu X., Dai X., Wang S., Shi X. (2019). A multiscale study of the penetration-enhancing mechanism of menthol. J. Tradit. Chin. Med. Sci..

[49] Trommer H., Neube R.H.H. (2006). Overcoming the stratum corneum: The modulation of skin penetration. Skin Pharmacol. Physiol..

[50] Prausnitz M.R., Gill H.S., Park J.-H. (2002). Microneedles for Drug Delivery. Modif. Release Drug Deliv..

[51] Ornelas J., Foolad N., Shi V., Burney W., Sivamani R.K. (2016). Effect of Microneedle Pretreatment on Topical Anesthesia: A Randomized Clinical Trial. Randomized Control. Trial.

[52] Arunkumar S., Shivakumar H.N., Murthy S.N. (2018). Effect of terpenes on transdermal iontophoretic delivery of diclofenac potassium under constant voltage. Pharm. Dev. Technol..

[53] Al-Mayahy M.H., Sabri A.H., Rutland C.S., Holmes A., Mckenna J., Marlow M., Scurr D.J. (2019). Insight into imiquimod skin permeation and increased delivery using microneedle pre-treatment. Eur. J. Pharm. Biopharm..

